# Autonomic function and signal complexity reflect level and severity of traumatic spinal cord injury

**DOI:** 10.1038/s41393-026-01285-z

**Published:** 2026-10-12

**Authors:** Modar Alhamdan, Linus Tegenborg Sjöström, Anders Hånell, Anders Lewén, Fartein Velle, Odin Joensen, Rozerin Kevci, David Fröjd Revesz, Mattias K. Sköld, Per Enblad, Teodor Svedung Wettervik

**Affiliations:** 1 Department of Medical Sciences, Section of Neurosurgery, Uppsala University, SE-751 85 Uppsala, Sweden; 2 Department of Neurosurgery, Uppsala University Hospital, SE-751 85 Uppsala, Sweden; 3 Department of Anaesthesiology and Intensive Care, Uppsala University Hospital, SE-751 85 Uppsala, Sweden; 4 Department of Neuroscience, Karolinska Institutet, SE-171 77 Stockholm, Sweden

**Keywords:** Prognostic markers, Trauma

## Abstract

**Study design:**

Observational single-centre cohort study.

**Objectives:**

To investigate heart rate variability (HRV), baroreflex sensitivity (BRS), and signal complexity (SC) and their relationships with neurological injury level and severity, as well as their prognostic value for six-month mortality and functional recovery in patients with traumatic spinal cord injury (TSCI).

**Setting:**

Uppsala University Hospital, Sweden.

**Methods:**

This study included 164 adult patients with TSCI treated in neurointensive care (NIC) (2008–2022). Continuous high-frequency physiological data from the first 7 post-injury days were analysed to derive HRV, BRS, and SC for heart rate (HR), mean arterial pressure, systolic blood pressure, and diastolic blood pressure (DBP). Associations with neurological injury level, injury severity, NIC physiology, neurological improvement, and six-month mortality were assessed.

**Results:**

Patients with cervical injuries had higher HRV, BRS (both p < 0.01), and SC for DBP (p < 0.05) than those with thoracic injuries. Complete cervical injuries had higher HRV (p < 0.05), but lower HR SC (p < 0.01) compared with incomplete cervical injuries. Higher HR SC was associated with neurological improvement at discharge (odds ratio 1.08 [95% confidence interval 1.01–1.16]; p < 0.05), whereas higher HRV was associated with increased six-month mortality (odds ratio 1.01 [95% confidence interval 1.00–1.02]; p < 0.05). Other autonomic and complexity measures showed limited prognostic value.

**Conclusion:**

Systemic autonomic indices after TSCI vary with injury level and severity and may capture distinct aspects of autonomic dysfunction beyond clinical grading. However, their limited prognostic value for neurological recovery and mortality suggest that outcome prediction after TSCI may depend more on other clinical factors and, possibly, more direct intraspinal physiological markers.

**Sponsorship:**

NA.

## Introduction

Traumatic spinal cord injury (TSCI) causes immediate primary damage to the spinal cord, but subsequent injury mechanisms substantially contribute to neurological deterioration and poor outcomes [1, 2]. Secondary injury mechanisms include vascular disruption, ischemia, and neuroinflammation, which develop within hours to days after the initial trauma [1–3]. In addition to acute decompression and stabilisation, neurointensive care (NIC) is essential to mitigate secondary injury, primarily through haemodynamic optimisation to maintain adequate spinal cord perfusion [4–6]. Current guidelines recommend maintaining mean arterial pressure (MAP) around at least 75–80 mmHg and not actively exceeding 90–95 mmHg for the first 3 to 7 days following injury, although evidence for specific thresholds remains limited [7, 8]. There is a complex dynamic interplay between systemic and spinal physiology, which may be disturbed after TSCI. Better knowledge about those mechanisms may be the clue to individualise treatment and refine prognostication.

Autonomic nervous system (ANS) dysfunction is common after TSCI, particularly in cervical and upper thoracic injuries, where damage to descending sympathetic pathways leads to loss of sympathetic tone and parasympathetic dominance [9–12]. Heart rate (HR) and arterial blood pressure (ABP) can be disrupted, leading to bradycardia, hypotension, orthostatic intolerance, and autonomic dysreflexia [13, 14]. Therefore ischaemia can develop in a sensitive and often swollen spinal cord post-injury, potentially causing irreversible secondary injury, increased cardiovascular morbidity, and worse outcome [11, 15, 16]. Heart rate variability (HRV) describes the variation in interbeat intervals and measures cardiac autonomic modulation [17, 18]. In TSCI, HRV is consistently reduced, reflecting the loss of sympathovagal balance [14, 19–23]. Similarly, baroreflex sensitivity (BRS), which indicates the heart rate response to spontaneous blood pressure fluctuations, is reduced after TSCI. Particularly in high-level spinal injuries, cardiovagal baroreflex function is markedly impaired [24–27]. Impaired BRS may contribute to cardiovascular complications, secondary insults, and unfavourable neurological outcome in this population [24–27].

Beyond traditional autonomic markers, signal complexity (SC) has emerged as a measure of physiological adaptability and regulatory capacity [28]. In simple terms, SC reflects how varied the fluctuations of a physiological signal are over time: a well-regulated system tends to generate complex fluctuations, whereas a system with exhausted regulatory reserves generates simpler and more regular signals [28]. Complex physiological signals may reflect intact regulatory mechanisms capable of responding to spontaneous fluctuations and physiological insults [28]. Hence, reduced SC may indicate exhausted compensatory reserves and a loss of adaptive capacity. Multiscale entropy quantifies SC, and reduced SC of intraspinal pressure has been suggested to correlate with secondary injury burden and worse outcomes in TSCI [29]. However, the SC of HR and ABP and the relationships between SC of systemic physiological signals and key NIC targets, such as ABP and HR remain virtually unexplored in TSCI. HRV, BRS, and SC may provide valuable information for fine-tuning of NIC and be a key to improve functional outcome.

The aim of this study was to explore HRV, BRS, and SC of systemic physiological signals (HR, systolic blood pressure [SBP], diastolic blood pressure [DBP], and MAP) in patients with severe TSCI during the first 7 days of NIC. Specifically, we aimed to investigate their relationships with neurological injury level and severity, as well as their prognostic value for functional recovery as assessed by the American Spinal Injury Association Impairment Scale (AIS) and six-month mortality. We hypothesised that ANS dysfunction and reduced SC would correlate with disturbed physiology and unfavourable recovery.

## Materials and methods

### Patients and study design

This observational, single-centre cohort study included all TSCI patients treated at the neurointensive care (NIC) unit, Uppsala University Hospital, Sweden, between 2008 and 2022. Three hundred and fourteen patients were identified with International Classification of Disease codes corresponding to a potential TSCI. The initial screening included a broad set of codes corresponding to spinal fractures and other unspecified spinal injuries (Supplementary table 1). The final study cohort consisted of 164 patients after exclusion of 10 patients aged less than 18 years at injury time, 112 patients with no actual traumatic spinal cord injury, 5 patients with conus/cauda equina injury (L1–5), 14 patients with no invasive ABP monitoring, and 9 patients with invasive ABP monitoring less than 24 h. Thus, the final cohort consisted of 164 patients (Supplementary figure 1).

### Management protocol

The management protocol for TSCI at the Department of Neurosurgery, Uppsala University Hospital, generally follows international guidelines for management of acute TSCI [7, 8, 30–32]. Treatment targets included SBP above around 120 mmHg, pO₂ >12 kPa, arterial glucose 5–10 mmol/L, haemoglobin ≥10 g/dL, electrolytes within normal limits, normovolaemia, and body temperature <38 °C. TSCI patients with respiratory insufficiency or co-occurring traumatic brain injury (TBI) typically required intubation and mechanical ventilation. In such cases, propofol was administered for sedation and morphine for analgesia, with additional analgesics/sedatives when needed, including paracetamol, clonidine, and gabapentin, titrated to clinical need. Neurological status examinations, including motor and sensory function, were conducted repeatedly in awake patients, and as more crude motor evaluations during wake-up tests in mechanically ventilated patients. Blood pressure was regulated primarily through intravenous administration of crystalloid fluids and albumin 200 mg/mL. When fluids alone were insufficient to achieve MAP targets, vasopressors (preferentially norepinephrine) were administered. Bradycardia was managed with atropine when clinically indicated. Early surgical decompression of the spinal cord and spinal fixation were performed when indicated and feasible, typically within 24 h of injury, via anterior, posterior, or combined approaches depending on the level and morphology of the injury.

### Data collection

Clinical variables were obtained through retrospective review of medical records. Demographics included age (years), sex (male/female), and Charlson Comorbidity Index (CCI) [33]. The AIS grade (scale) at admission to NIC unit, ranging from A (complete sensory and motor impairment below the level of injury) to E (normal neurological function), was assessed by neuro-specialised physiotherapists according to the American Spinal Injury Association’s and International Spinal Cord Society’s International Standards for Neurological Classification of Spinal Cord Injury [34]. The neurological level of injury (high [C1–4]/low [C5–8] cervical or high [T1–6]/low [T7–12] thoracic) and severity (complete/incomplete) at admission were recorded. Complete injuries were defined as AIS grade A at admission to the NIC unit, while incomplete injuries as AIS grade other than A. The time between injury and admission (days) was noted. Medications during NIC were recorded, including vasopressors during NIC (yes/no) and analgesics (yes/no). Duration of NIC (days), defined as time from admission to the NIC unit until discharge, and percentage of NIC time spent in mechanical ventilation (%) were documented. Monitoring time (hours) of ECG and ABP were also recorded. Autonomic dysreflexia (yes) was identified as an episode of hypertension accompanied by bradycardia and flushing above the level of the lesion.

Functional outcome was measured as neurological improvement at discharge (yes/no) and at long-term follow-up (yes/no), defined as a better AIS grade at discharge and long-term follow-up, respectively, compared to AIS grade at admission to NIC unit. Mortality (yes/no) at 1 and 6 months was documented as well. Timepoint of follow-up (months) was registered.

Physiological signals, including ABP and HR were prospectively collected through the Odin software [35]. ABP was sampled at 100 Hz, ECG at 200 Hz. ABP was measured using an arterial line at heart level. Data were analysed for the first 7 days (168 h) of NIC counting from the time-point of injury.

### Data analysis

Heartbeats were identified using a moving-average QRS detector adapted for continuous NIC monitoring [36]. The ECG signal was bandpass-filtered and squared to enhance QRS complexes. Dual moving-average filters were applied to separate QRS complexes from the background cardiac rhythm, and R-peaks were localised as local maxima within detected regions. A refractory period of 300 ms was enforced to prevent double detections. Interbeat intervals (i.e., NN intervals) were calculated in seconds as the time difference between consecutive R-peaks [17, 18]. Beat-to-beat heart rate (beats per minute [BPM]) was computed as 60 seconds divided by each interbeat interval. Beat-to-beat systolic blood pressure (SBP) was defined as the maximum ABP value between two consecutive R-peaks. Beat-to-beat diastolic blood pressure (DBP) was defined as the minimum ABP value between two consecutive R-peaks. Beat-to-beat MAP was calculated as the mean ABP value between two consecutive R-peaks.

The valid monitoring time (VMT) was defined as the cumulative period with valid data points after excluding periods without valid data, e.g., artefacts or during surgery or radiological imaging. To quantify the burden of physiological insults percentages of VMT (%VMT) with physiological variables beyond predefined thresholds were calculated. The %VMTs of MAP below 80 mmHg and SBP below 120 mmHg were calculated to reflect the proportion of time BP was below treatment targets in the current guidelines for TSCI [7, 8]. Physiologically implausible values, defined as values outside the ranges interbeat intervals 0.25–3.0 seconds (corresponding to HR 20–240 BPM), SBP 50–250 mmHg, MAP 30–200 mmHg, and DBP 20–150 mmHg were automatically excluded as artefacts. All data processing and analyses were conducted in RStudio (version 2025.09.0).

#### Heart rate variability

Time-domain HRV was analysed for the first 7 days (168 h) of NIC in accordance with the standard recommendations of the Task Force of the European Society of Cardiology and the North American Society of Pacing and Electrophysiology [17, 18, 37]. The derived indices were SDNN (standard deviation of all normal-to-normal [NN] intervals [ms]) and RMSSD (root mean square of successive differences between adjacent NN intervals [ms]). SDNN reflects overall HRV and is modulated by sympathetic and parasympathetic activity, whereas RMSSD characterises beat-to-beat variability and is predominantly influenced by vagal modulation [18].

#### Baroreflex sensitivity

BRS was calculated using the sequence method for the first 7 days of NIC [38–40]. Each beat-to-beat SBP value was paired with the immediately preceding interbeat interval from the same cardiac cycle (0-beat delay). Non-overlapping sequences of at least 3 adjacent beats, in which SBP and interbeat interval changed concordantly in the same direction, were identified. Pearson’s correlation between SBP and interbeat interval was computed for each candidate sequence. A sequence was considered valid if the correlation was statistically significant (p < 0.05) and the SBP difference across the sequence exceeded 2.5 mmHg. No minimum interbeat interval variation was required. BRS (ms/mmHg) for each sequence was defined as the slope of the corresponding regression line. Patient BRS was expressed as the median across all valid sequence values.

#### Multiscale entropy

Multiscale entropy quantifies SC across multiple time scales [28, 41–43], and was analysed for the first 7 days of NIC following established methods [28, 41–44], to derive the SC-index for HR, MAP, SBP, and DBP. In short, raw data for each variable were averaged within non-overlapping 10-second windows (Scale 1). Every 2 consecutive Scale 1 values were averaged to construct Scale 2, and each 3 consecutive Scale 1 values were averaged to form Scale 3. Twenty timescales were generated in the same manner for each variable. Sample Entropy (SE) was computed at each timescale using an embedding dimension (m = 2) and a tolerance coefficient (r = 0.15 × standard deviation for the respective scale). SE was defined as the negative logarithm of the conditional probability that two sequences of length (m) similar within tolerance (r) remain similar at the subsequent point. SC-index was calculated as the area under the SE curve across all 20 timescales.

### Statistical analyses

Categorical variables were presented as numbers (percentages) and numerical variables as medians (interquartile range [IQR]). Spearman’s correlation test was used to assess the associations between SDNN, RMSSD, BRS, and SC of HR, MAP, SBP, and DBP vs. demographics (age, sex, and CCI), NIC characteristics (days in NIC, percentage of NIC time spent in mechanical ventilation, vasopressor use, and analgesic use), and key physiological NIC variables (MAP, SBP, DBP, and HR). The effects of demographic, physiological, HRV, BRS, and SC variables on each of neurological improvement at discharge (yes/no) and mortality at 6 months (yes/no) were examined using univariable binomial logistic regression. To account for confounder effects, separate multivariable binomial logistic regression analyses were performed for SDNN, RMSSD, BRS, and SC variables vs. neurological improvement at discharge (yes/no) and mortality at 6 months (yes/no), adjusted for age (years), AIS at admission (grade), neurological level of injury (high cervical C1-4, low cervical C5-8, high thoracic Th1-6, low thoracic Th7-12), and percentage of neurointensive-care time on mechanical ventilation. Similar univariable and multivariable regression analyses were performed for patients with cervical injuries as sensitivity analyses. Odds ratios were presented per 1 unit. Subgroup analyses comparing cervical vs. thoracic injuries, complete vs. incomplete cervical injuries, and high (C1–4) vs. low (C5–8) cervical injuries were performed to assess the effect of the neurological level and severity of the injury on SC and ANS. Statistical significance was tested using Mann-Whitney U test for continuous and Chi-squared test for categorical variables. A p-value below 0.05 was considered statistically significant. Since this was an explorative study, all p-values were reported unadjusted. All statistical analyses and visualisations were performed in RStudio (version 2025.09.0).

### Data visualisation

Single-variable heatmaps were generated to illustrate the relationship between SDNN, RMSSD, BRS, and SC-index metrics (HR, MAP, SBP, DBP) vs. outcome over the first 7 days of NIC after injury. SDNN, RMSSD, BRS, and SC-index of HR, MAP, SBP, and DBP were derived across non-overlapping 4-hour windows (≥75% VMT; minimum 3 h). These calculations across shorter time frames were performed to explore the temporal dynamics of these variables across the observation period, rather than analysing on aggregated value per variable for the whole period. The 4-hour window was chosen because complexity calculations require at least 50 observations at the 20th timescale, equivalent to around 2.8 h of monitoring time at the raw timescale [28]. The range of each variable was divided into 10 bins. For instance, for SC-index ranging between 0–30, the first bin was between 0–3, the second between 3–6, etc. For each patient, %VMT of the observation period spent within each bin was calculated. The %VMT was then correlated with neurological improvement at discharge (yes/no) using Spearman’s rank correlation, producing a single ρ coefficient per bin. Correlation coefficients were expressed using a colour gradient, where blue indicated association with neurological improvement and red with lack of improvement. Bins containing data from fewer than 5 patients with at least 4 h of monitoring were excluded and displayed as white. Finally, each bin was subdivided into 5 sub-bins and Gaussian kernel smoothing was applied with a standard deviation of 4 bins. Complementary density heatmaps were created to show the normalised distribution of time spent within each variable range across patients. For each bin, %VMT was calculated per patient, across all patients, and normalised to a 0 (red) – 1 (blue) scale. Furthermore, generalised additive model (GAM) plots were created to illustrate the temporal dynamics of SC across the first 7 NIC days post-injury stratified for neurological improvement at discharge (yes/no). The plots combined each 4-hour data point of SC-index variables (HR, MAP, SBP, and DBP) with time since injury.

## Results

### Demographics and baseline characteristics

A total of 164 TSCI patients were included. Median age was 65 (47–74) years, 24% were female, and median CCI was 2 (0–4; Table 1). Most patients had cervical injuries (84%). Half of the patients (50%) presented with AIS A at admission. During NIC, 74% received vasopressors and 99% analgesics.

**Table 1:** Demographics, injury, treatment, and outcome.

Variable	N (%) or median (IQR)
Patients	164 (100%)
Demography	
Age (years)	65 (47–74)
Sex (female)	40 (24%)
Charlson comorbidity index (scale)	2 (0–4)
Injury	
*AIS at admission*	
A	82 (50%)
B	16 (10%)
C	49 (30%)
D	15 (9%)
E	1 (0.6%)
Severity of injury (complete/incomplete)	82 (50%)/81 (49%)
*Neurological level of injury*	
High cervical (C1–4)	36 (22%)
Low cervical (C5–8)	102 (62%)
High thoracic (Th1–6)	16 (10%)
Low thoracic (Th7–12)	9 (6%)
Time from injury to admission (days)	1 (0–1)
Medications during NIC	
Vasopressors (yes)	122 (74%)
Analgesics (yes)	163 (99%)
Outcome	
Timepoint of follow-up (months)	8 (3–42)
Neurological improvement at discharge (yes)	38 (23% of 163)
Neurological improvement at long-term follow-up (yes)	52 (55% of 94)
Mortality 30 days post-injury (yes)	11 (7%)
Mortality 6 months post-injury (yes)	27 (16%)

Missing: AIS at admission = 1. Severity of injury = 1. Neurological level of injury = 1. Neurological improvement at discharge = 1. Neurological improvement at long-term follow-up = 70. The patient with AIS at admission E had clear spinal cord oedema meaning that the spinal cord was affected but motor and sensory functions were intact, it is therefore not a clear E, but the patient could not be excluded due to spinal cord oedema.

*IQR*, interquartile range; *AIS*, American Spinal Injury Association Impairment Scale; *NIC*, neurointensive care; *TBI*, traumatic brain injury.

### Physiological characteristics during NIC

The median time of the total NIC stay was 8 (4–14) days, and patients were mechanically ventilated for a median of 50 (0–100) % of NIC time (Table 2). Median MAP was 81 (76–85) mmHg, falling below 80 mmHg in 45 (28–63) % of VMT. Median SBP was 130 (124–138) mmHg, with SBP below 120 mmHg in 29 (17–43) % of VMT. Median DBP was 59 (55–62) mmHg and median HR was 71 (64–81) BPM.

**Table 2:** Monitoring time and physiological characteristics during NIC.

Variables	Median (IQR) or n (%)
Time of NIC (days)	8 (4–14)
Mechanical ventilation during NIC (%)	50 (0–100)
Monitoring time of ECG (hours)	156 (146–162)
Monitoring time of ABP (hours)	153 (130–160)
Autonomic dysreflexia during NIC (yes)	7 (4%)
MAP (mmHg)	81 (76–85)
MAP < 80 mmHg (%VMT)	45 (28–63)
SBP (mmHg)	130 (124–138)
SBP < 120 mmHg (%VMT)	29 (17–43)
DBP (mmHg)	59 (55–62)
HR (bpm)	71 (64–81)
SDNN (ms)	125 (101–162)
RMSSD (ms)	56 (37–88)
BRS (ms/mmHg)	4.4 (3.2–6.7)
HR SC-index	11 (8–15)
MAP SC-index	16 (14–19)
SBP SC-index	16 (13–19)
DBP SC-index	15 (12–18)

The reported data is aggregated across the whole observation period.

*NIC*, neurointensive care; *IQR*, interquartile range; *ECG*, electrocardiography; *ABP*, arterial blood pressure; *MAP*, mean arterial pressure; *SBP*, systolic blood pressure; *DBP*, diastolic blood pressure; *HR*, heart rate; *bpm*, beats per minute; *%VMT*, percentage of valid monitoring time; *SDNN*, standard deviation of all NN intervals; *ms*, milliseconds; *RMSSD*, root mean square of adjacent NN interval differences; *BRS*, sequence baroreflex sensitivity; *SC-index*, signal complexity index.

### Heart rate variability, baroreflex sensitivity, and multiscale entropy

Median SDNN was 125 (101–162) ms, RMSSD 56 (37–88) ms, and BRS 4.4 (3.2–6.7) ms/mmHg (Table 2). Regarding SC-index, median values were 11 (8–15) for HR, 16 (14–19) for MAP, 16 (13–19) for SBP, and 15 (12–18) for DBP.

#### Correlation with neurological level and severity of injury

Subgroup analyses by injury level and severity are presented in Table 3. Compared to patients with thoracic spinal injuries (n = 25), patients with cervical spinal injuries (n = 138) had significantly higher SDNN (92 vs. 129 ms; p < 0.001), RMSSD (39 vs. 59 ms; p < 0.01), BRS (3.6 vs. 5.1 ms/mmHg; p < 0.01), and DBP SC-index (14 vs. 15; p < 0.05), as well as a greater proportion of NIC time spent in mechanical ventilation (5% vs. 50%; p < 0.05). SC-index of HR, MAP, and SBP did not differ significantly between cervical and thoracic spinal injuries. Patients with high (C1–4) cervical spinal injury spent a significantly higher proportion of NIC time in mechanical ventilation (100% vs. 36%; p < 0.0001) and had higher SDNN (140 vs. 124; p < 0.05) compared to patients with low (C5–8) injuries.

**Table 3:** Subgroup analyses: cervical vs. thoracic and complete vs. incomplete injuries.

Variable	Median (IQR) or n (%)		p-value	Median (IQR) or n (%)		P-value
	Cervical	Thoracic		Cervical		
				Complete	Incomplete	
Patients	138 (100%)	25 (100%)	–	64 (46%)	74 (54%)	–
Physiology during NIC						
MAP (mmHg)	81 (76–86)	80 (75–84)	0.270	79 (76–83)	83 (77–88)	***0.0109***
SBP (mmHg)	130 (124–138)	130 (122–137)	0.453	129 (123–133)	131 (126–144)	***0.0156***
DBP (mmHg)	60 (55–62)	58 (54–61)	0.718	58 (56–62)	59 (53–64)	0.947
HR (bpm)	70 (64–80)	81 (69–92)	***0.00424***	70 (63–78)	72 (65–81)	0.364
Outcome						
Neurological improvement at discharge (yes)	29 (21%)	9 (36%)	0.103	11 (17%)	18 (24%)	0.305
Neurological improvement at long-term follow-up (yes)	45 (57%)	7 (47%)	0.462	19 (51%)	26 (62%)	0.344
Mortality 30 days post-injury (yes)	10 (7%)	1 (4%)	0.684	6 (9%)	4 (5%)	0.526
Mortality 6 months post-injury (yes)	24 (17%)	3 (12%)	0.582	11 (17%)	13 (18%)	0.953
Autonomic nervous system and mechanical ventilation						
Mechanical ventilation during NIC (%)	50 (0–100)	5 (0.0–67)	***0.0342***	81 (46–100)	33 (0–74)	***0.000107***
SDNN (ms)	129 (106–170)	92 (75–132)	***0.000742***	141 (113–195)	121 (102–149)	***0.00242***
RMSSD (ms)	59 (40–94)	39 (29–57)	***0.00576***	69 (48–116)	54 (38–76)	0.0768
BRS (ms/mmHg)	5.1 (3.4–6.8)	3.6 (2.7–4.0)	***0.00147***	5.7 (3.4–7.3)	4.4 (3.2–6.7)	0.241
HR SC-index	11 (8–14)	14 (8–16)	0.209	10 (7–12)	13 (8–17)	***0.00198***
MAP SC-index	16 (14–19)	14 (13–18)	0.140	16 (14–18)	16 (14–19)	0.499
SBP SC-index	16 (14–19)	16 (13–21)	0.756	16 (13–18)	16 (14–19)	0.321
DBP SC-index	15 (13–18)	14 (11–16)	***0.0425***	15 (13–18)	15 (12–18)	0.927

Subgroup analyses of complete vs. incomplete injuries were done in patients with cervical injuries. Missing: Neurological improvement at discharge = 1. Neurological improvement at long-term follow-up = 70.

The reported data is aggregated across the whole observation period.

*IQR*, interquartile range; *NIC*, neurointensive care; *MAP*, mean arterial pressure; *SBP*, systolic blood pressure; *DBP*, diastolic blood pressure; *HR*, heart rate; *bpm*, beats per minute; *SDNN*, standard deviation of all NN intervals; *ms*, milliseconds; *RMSSD*, root mean square of adjacent NN interval differences; *BRS*, sequence baroreflex sensitivity; *SC-index*, signal complexity index; *HR*, heart rate; *MAP*, mean arterial pressure; *SBP*, systolic blood pressure; *DBP*, diastolic blood pressure.

Compared to patients with incomplete cervical TSCI (n = 74), patients with complete cervical injuries (n = 64) showed significantly higher SDNN (121 vs. 141 ms; p < 0.01) and a higher proportion of NIC time in mechanical ventilation (33% vs. 81%; p < 0.001), but lower HR SC-index (13 vs. 10; p < 0.01). RMSSD, BRS, and SC-index of MAP, SBP, and DBP did not differ significantly between complete and incomplete cervical injuries.

#### Correlation with baseline characteristics and NIC targets

**Fig. 1:**
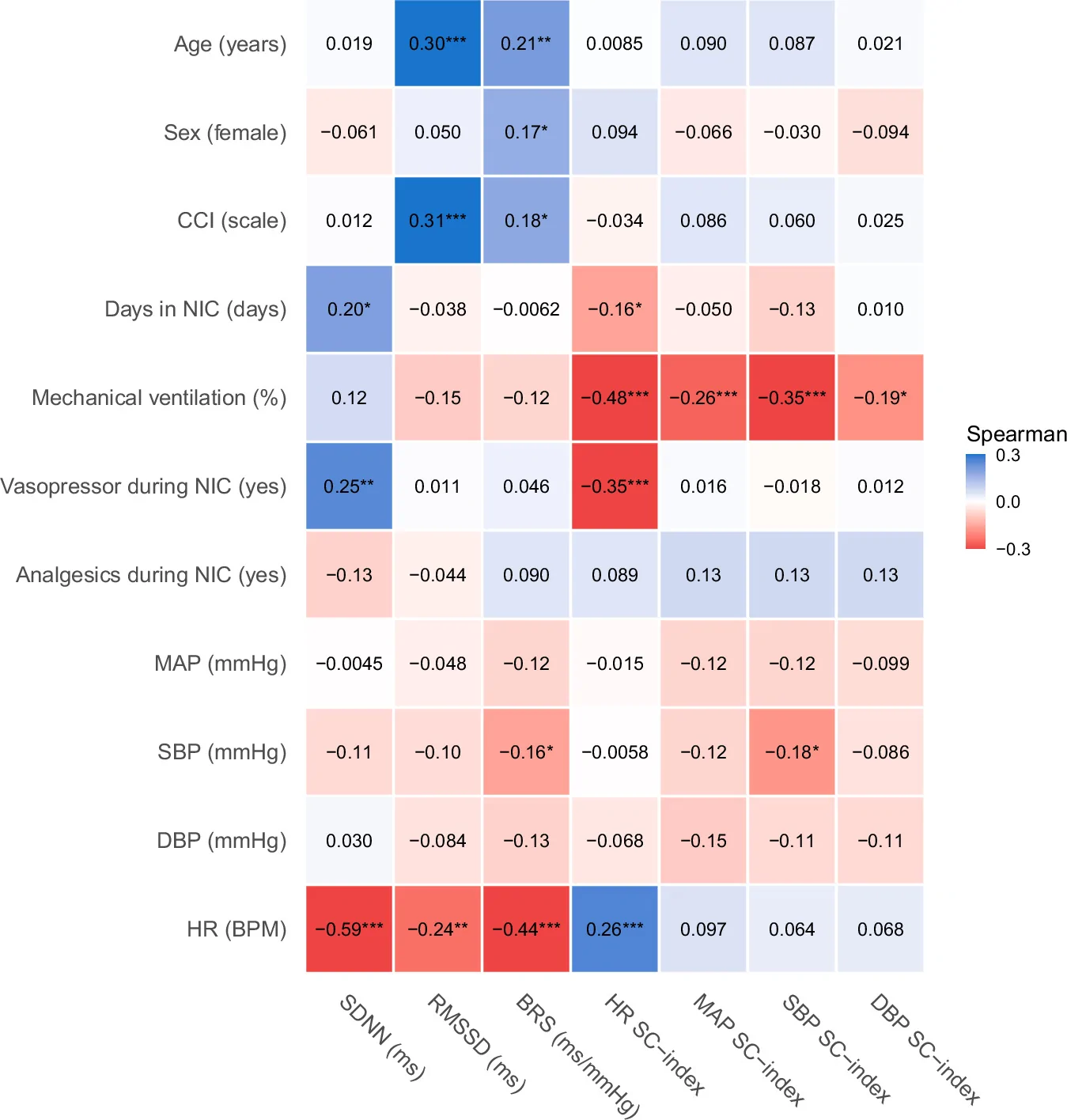
Spearman’s correlation: HRV, BRS, and SC vs. baseline characteristics and physiological variables. P-value below 0.05 was considered statistically significant. * p < 0.05, ** p < 0.01, *** p < 0.001. Exact p-values are presented in Supplementary table 2. Each grid cell contains the exact Spearman’s correlation coefficient (ρ). The colour of the grid cell corresponds to ρ; numerically positive correlations are coloured blue and negative red. CCI = Charlson Comorbidity Index. NIC = neurointensive care. Mechanical ventilation (%) = percentage of NIC time spent in mechanical ventilation. MAP = mean arterial pressure. SBP = systolic blood pressure. DBP = diastolic blood pressure. HR = heart rate. BPM = beats per minute. SDNN = standard deviation of all NN intervals. RMSSD = root mean square of adjacent NN interval differences. BRS = sequence baroreflex sensitivity. SC-index = signal complexity index.

Spearman’s correlation analyses between baseline characteristics and NIC physiology vs. HRV, BRS, and SC are presented in Fig. 1. Older age and higher CCI were associated with higher RMSSD (ρ 0.30 and ρ 0.31; both p < 0.001) and BRS (ρ 0.21; p < 0.01 and ρ 0.18; p < 0.05). Female sex was associated with higher BRS (ρ 0.17; p < 0.05). Longer NIC time correlated with higher SDNN (ρ 0.20; p < 0.05) and lower HR SC-index (ρ −0.16; p < 0.05). A greater proportion of NIC time in mechanical ventilation was associated with lower SC across all variables (HR ρ −0.48; MAP ρ −0.26; SBP ρ −0.35; all p < 0.001; DBP ρ −0.19; p < 0.05). Vasopressor administration during NIC was linked to higher SDNN (ρ 0.25; p < 0.01) and lower HR SC-index (ρ −0.35; p < 0.001).

Regarding NIC physiological targets, higher HR was associated with lower SDNN (ρ −0.59; p < 0.001), RMSSD (ρ −0.24; p < 0.01), and BRS (ρ −0.44; p < 0.001), but higher HR SC-index (ρ 0.26; p < 0.001). Higher SBP correlated with lower BRS (ρ −0.16; p < 0.05) and lower SBP SC-index (ρ −0.18; p < 0.05). Exact p-values are presented in Supplementary Table 2.

### Outcome

**Fig. 2:**
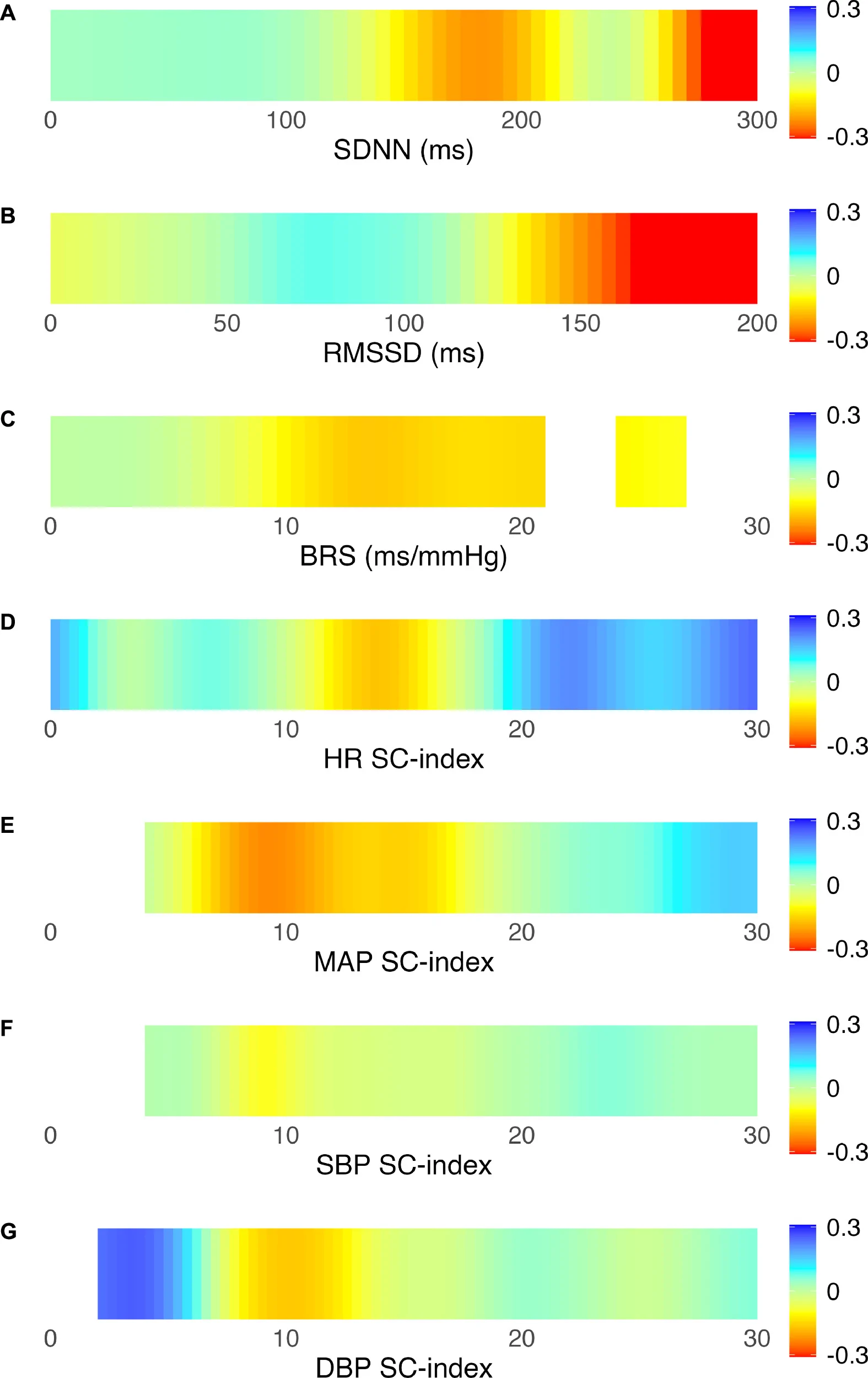
Heart rate variability, baroreflex sensitivity, signal complexity vs. neurological improvement at discharge (yes/no). Panels **A–G** show heatmaps of correlations of heart rate variability, baroreflex sensitivity, and signal complexity with neurological improvement at discharge. Spearman correlations were performed for %VMT in each bin vs. neurological improvement at discharge (yes/no). Blue cells denote association with neurological improvement at discharge, whereas red cells denote association with no neurological improvement at discharge. SDNN = standard deviation of all NN intervals. RMSSD = root mean square of successive differences between adjacent NN intervals. BRS = baroreflex sensitivity. HR = heart rate. SC-index = signal complexity index. MAP = mean arterial pressure. SBP = systolic blood pressure. DBP = diastolic blood pressure. %VMT = percentage of valid monitoring time.

**Fig. 3:**
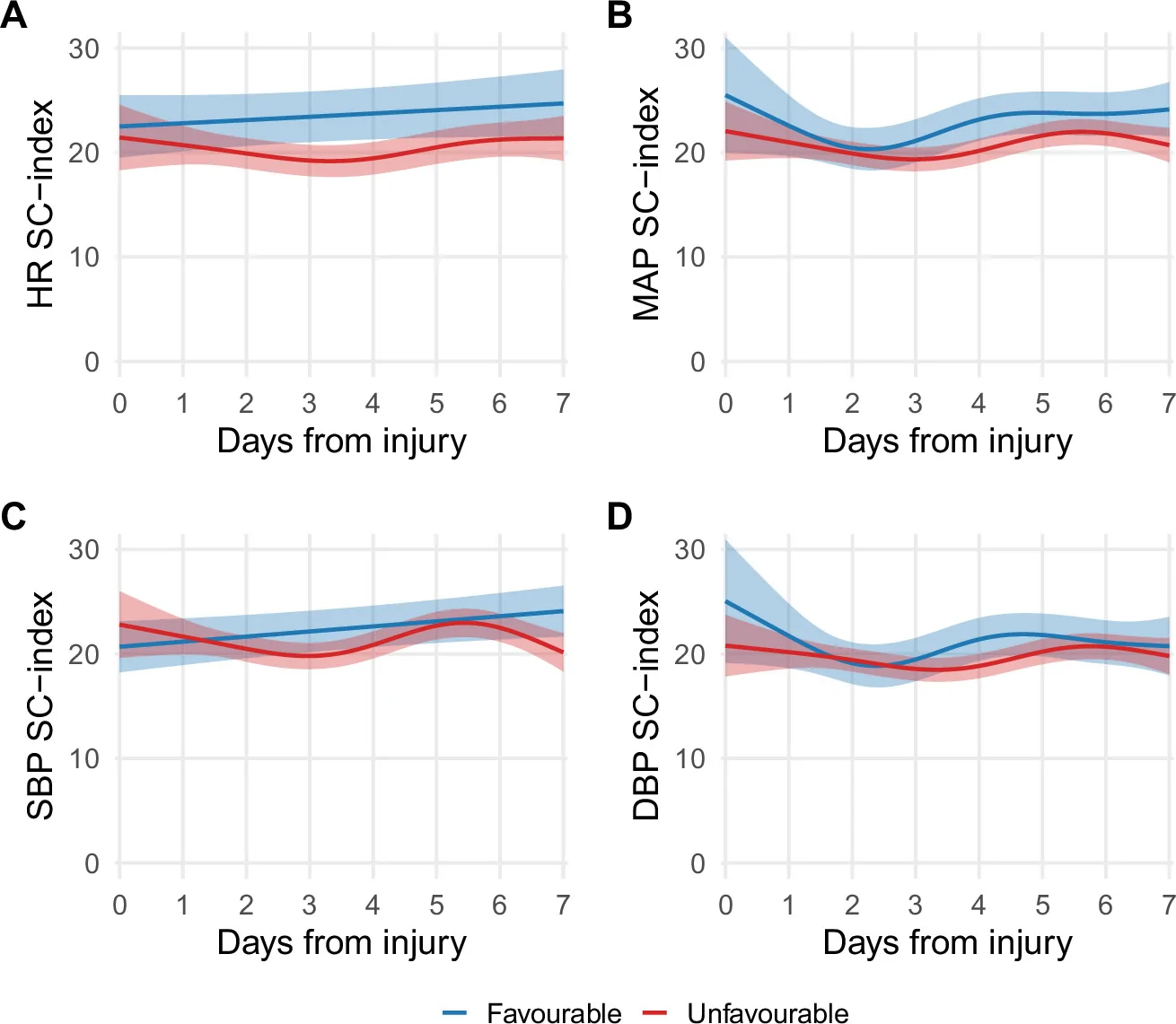
GAM plots of signal complexity vs. time stratified for outcome at discharge. Panels **A–D** show GAM plots for SC variables across the 7-day observation period stratified for outcome at discharge. Blue represent patients with neurological improvement at discharge (favourable outcome), and red patients with no improvement at discharge (unfavourable outcome). GAM = generalised additive model. HR = heart rate. SC-index = signal complexity index. MAP = mean arterial pressure. SBP = systolic blood pressure. DBP = diastolic blood pressure.

At discharge (8 [4–14] days), 23% (of 163 available) had achieved neurological improvement and 55% (of 94 available) at a median follow-up time of 8 (3–42) months. Mortality was 7% at 30 days and 16% at 6 months (Table 1). In univariable binomial logistic regression analyses (Table 4), higher HR SC-index was associated with improvement (OR 1.08; p < 0.05) and higher RMSSD (OR 1.01; p < 0.05) correlated with increased mortality at 6 months. These associations persisted after exclusion of patients with thoracic injuries (Supplementary table 3). Other HRV, BRS, and SC variables were not significantly associated with neurological improvement at discharge or six-month mortality. HRV, BRS, or SC variables were not independently associated with outcome after adjustment for age, AIS at admission, neurological level of injury, and mechanical ventilation time (Supplementary table 4). The relationships between HRV, BRS, and SC and neurological improvement at discharge were further illustrated in single-variable heatmaps (Fig. 2), with corresponding density heatmaps showing the distribution of observations across variable ranges (Supplementary figure 2). Temporal dynamics of SC during the first 7 days of NIC following injury and stratified for outcome at discharge are shown in GAM plots stratified by neurological improvement (Fig. 3). SC variables varied during the 7 day period. Complementary temporal GAM plots of raw haemodynamics stratified for outcome at discharge are presented in Supplementary figure 3. Outcome did not differ significantly between patients with complete and incomplete cervical injuries (Table 3). However, high (C1–4) cervical injuries had a significantly higher 30-day mortality rate (19% vs. 2.9%; p < 0.01) compared to lower (C5–8) cervical injuries.

**Table 4:** Univariable binomial logistic regression: physiological variables vs. neurological improvement at discharge (yes/no) and mortality at 6 months (yes/no).

Variables	Improvement at discharge (yes)		Mortality at 6 months (yes)	
	OR (95% CI)	p-value	OR (95% CI)	p-value
Age (years)	0.99 (0.97–1.01)	0.431	1.09 (1.05–1.15)	***<0.0001***
Charlson Comorbidity Index (scale)	0.89 (0.73–1.08)	0.244	1.79 (1.42–2.33)	***<0.0001***
Time in NIC (days)	1.00 (0.96–1.03)	0.957	0.95 (0.88–1.00)	0.128
Mechanical ventilation during NIC (%)	0.98 (0.97–0.993)	***<0.0001***	1.01 (1.00–1.02)	***0.0200***
SDNN (ms)	0.99 (0.99–1.00)	0.113	1.00 (0.99–1.01)	0.832
RMSSD (ms)	0.99 (0.98–1.00)	0.0527	1.01 (1.00–1.02)	***0.0253***
BRS (ms/mmHg)	1.00 (0.90–1.08)	0.909	1.03 (0.93–1.12)	0.576
HR SC-index	1.08 (1.01–1.16)	***0.0185***	0.95 (0.88–1.03)	0.234
MAP SC-index	1.03 (0.94–1.13)	0.588	1.02 (0.92–1.14)	0.652
SBP SC-index	1.05 (0.97–1.14)	0.251	0.95 (0.85–1.04)	0.281
DBP SC-index	1.00 (0.91–1.09)	0.982	1.00 (0.90–1.11)	0.949

Odds ratios are presented per 1 unit. P-value below 0.05 was considered statistically significant. Missing: For improvement at discharge: 1.

*SDNN*, standard deviation of all NN intervals; *ms*, milliseconds; *RMSSD*, root mean square of adjacent NN interval differences; *BRS*, sequence baroreflex sensitivity; *HR*, heart rate; *SC-index*, signal complexity index; *MAP*, mean arterial pressure; *SBP*, systolic blood pressure; *DBP*, diastolic blood pressure.

## Discussion

Disturbance of autonomic cardiovascular control is increasingly recognised as an important component of SCI pathophysiology, yet its temporal dynamics and clinical significance remain incompletely understood. This observational study investigated HRV, BRS, and SC of systemic physiological signals using continuous high-frequency monitoring in 164 patients with TSCI during NIC. Patients with cervical injuries had higher HRV, BRS, and SC of DBP than those with thoracic injuries, and complete cervical injuries showed higher SDNN but lower SC of HR than incomplete cervical injuries. Higher SC of HR correlated with neurological improvement at discharge, while higher RMSSD was associated with increased six-month mortality. In contrast to previously reported reductions in HRV after TSCI [14, 19–23], the absolute values in this cohort were numerically within reported ranges in healthy individuals [45]. This indicates sympathovagal imbalance and altered autonomic function in TSCI with parasympathetic predominance and sympathetic dysfunction, which can be masked as numerically higher autonomic indices. Still, consistent with previous SCI studies [24–27], BRS was disrupted in this cohort.

Altogether, our findings suggest that autonomic indices vary significantly depending on level and severity of TSCI. Thus, these indices may potentially contribute to the bedside assessment of autonomic function, beyond level and severity of injury. However, in contrast to our hypothesis and findings across various acute brain injury conditions [41–44, 46–48], HRV, BRS, and SC demonstrated very limited prognostic value for neurological improvement and mortality in TSCI. Hence, outcome after TSCI may be affected by other variables, such as pre-injury status, the severity of the primary SCI, other injuries, or intraspinal physiology and SC.

### Heart rate variability, baroreflex sensitivity, and signal complexity

The preserved SDNN and RMSSD combined with reduced BRS in this cohort do not corroborate the notion of globally depressed autonomic modulation following TSCI [14, 19–23]. This finding can be confounded by the observation period, as HRV indices may vary depending on the length of the observation period [17]. Still, these findings are coherent with the neuroanatomy of cardiovascular autonomic control after TSCI. Cervical spinal injuries interrupt descending sympathetic pathways to the heart, while supraspinal parasympathetic control via the vagal nerve remains intact [9, 10, 12–14]. The result can be unopposed parasympathetic drive of the sinoatrial node, manifesting as preserved SDNN (driven by both sympathetic and vagal modulation) and RMSSD (vagally driven beat-to-beat variability) [18]. Regarding SC, although absolute SC values cannot be directly compared with the literature, due to methodological differences, a previous study has demonstrated that SC of intraspinal pressure was disrupted following TSCI [29]. Furthermore, SC variables varied during the seven-day post-injury period in the GAM plots, suggesting that systemic SC dynamically evolves during the first 7 days after injury in TSCI, rather than being established at the time of injury. Altogether, autonomic function seems to be altered following TSCI suggesting sympathovagal imbalance.

### Influence of injury and clinical characteristics

Patients with cervical injuries displayed significantly higher SDNN, RMSSD, BRS, and SC of DBP than those with thoracic injuries, despite a significantly greater proportion of NIC spent in sedation and mechanical ventilation in the cervical group, which would be expected to suppress autonomic variability [37]. This is consistent with prior reports that cardiovascular autonomic control after TSCI is dependent on the neurological level of the injury, but it contrasts with the notion that higher-level SCI uniformly reduces autonomic variability [14, 19–23]. This suggests that the preserved autonomic indices in cervical TSCI do not necessarily reflect preserved autonomic function, but rather sympathovagal imbalance with parasympathetic predominance and disrupted descending sympathetic pathways. Similarly, complete cervical injuries had significantly higher SDNN than incomplete cervical injuries, but significantly lower SC of HR. This dissociation suggests that conventional HRV indices and SC may capture different aspects of autonomic control, as SDNN and RMSSD quantify the RR-interval fluctuations, while SC of HR reflects rather non-linear temporal patterns of these fluctuations. Hence, complete cervical injuries may show greater variability possibly reflecting unopposed vagal modulation following more profound disruption of descending sympathetic pathways, while incomplete cervical injuries may retain more complex cardiovascular regulation. Thus, in acute TSCI, autonomic indices seem to reflect sympathovagal imbalance related to injury level and severity rather than physiological compensatory capacity, which corroborates conclusions of previous studies [14, 20]. Autonomic indices seem generally sensitive for injury level and severity, thus potentially contributing to the bedside assessment of autonomic function, beyond level and severity of injury.

Older age and higher CCI were associated with higher RMSSD and BRS, contrary to established observation of reduced HRV in older patients [18, 37], which supports the idea of increased HRV in this cohort reflecting sympathovagal imbalance rather than healthy variability. Additionally, higher HR correlated with lower SDNN, RMSSD, and BRS, likely reflecting the inverse correlation between sympathetic modulation increasing HR and decreasing HRV indices and parasympathetic drive [18, 37]. However, HR can also be confounded by factors like pain, sedation, vasopressors, and mechanical ventilation. In this cohort, a greater proportion of NIC time spent in sedation and mechanical ventilation correlated with reduced SC across all variables, presenting a confounder for potentially reduced SC. Lastly, higher HR (likely driven by sympathetic modulation) was significantly associated with more complex signal of HR, suggesting that SC of HR may be more preserved in patients with preserved sympathetic drive.

### Outcome

Autonomic function, including HRV, BRS, and SC, demonstrated very limited prognostic value for neurological improvement at discharge and mortality at six months following TSCI, which is consistent with the scarce literature reporting poor prognostic value of HRV in general SCI cohorts [49]. Higher SC of HR significantly correlated with neurological improvement at discharge in this cohort, consistent with the brain injury literature, in which greater complexity reflects intact regulatory capacity capable of compensating for spontaneous fluctuations and pathological physiology [41–44]. Additionally, higher RMSSD was significantly associated with six-month mortality, further supporting the notion that the preserved autonomic indices in this cohort may represent sympathovagal imbalance with parasympathetic predominance rather than healthy physiological compensation. In temporal GAM plots, SC varied throughout the 7 day observation period, suggesting that physiological SC changes dynamically following TSCI, rather than being a base value determined at the time of injury.

The limited prognostic information observed in this cohort contrasts the consistent predictive value of HRV and SC in acute brain injuries [41–44, 46–48]. In brain injury cohorts, intracranial SC – compared to systemic – is particularly associated with outcome [41–44]. Similarly, SC of intraspinal pressure has been reported to significantly predict outcome following TSCI [29]. Thus, the extraspinal SC reported in the current study may not capture pathological patterns that provide prognostic information as well as intraspinal SC. Compared to acute brain injury, the focal neuroanatomical nature of TSCI may contribute to the lack of prognostic information in systemic SC. Hence, functional outcome following TSCI may mainly be affected by other variables than autonomic indices, such as pre-injury status, primary TSCI, and time to surgery, but it cannot be excluded that the overall NIC management may have tempered the disturbances in autonomic function to some extent and reduced the impact on functional outcome.

### Methodological considerations

The main strength of this study is the large cohort of TSCI patients (n = 164) with high-frequency physiological NIC data and granular clinical and radiological records, where HRV, BRS, and SC of systemic physiological signals were explored. Still, the study has several limitations. First, it is limited by its retrospective, observational, single-centre design. Although the cohort (n = 164) is relatively large in the TSCI context, the subgroup of thoracic spinal injuries was small (n = 25), which limits the statistical power of comparisons between cervical and thoracic spinal injuries and increases the risk of type I and II errors. Second, patients were sedated and mechanically ventilated in a median of 50 (0–100) % of NIC time, which is known to affect autonomic function [37]. Thus, sedation, mechanical ventilation, and various pharmacological interventions including cardiovascular active agents, present important confounders in this cohort, particularly in patients with cervical and/or complete injuries who were sedated and ventilated to a greater extent compared to thoracic and/or incomplete injuries. However, autonomic indices were increased rather than decreased in the groups with greater proportions of NIC time spent in sedation and mechanical ventilation. Third, the univariable regression analyses exploring outcome associations were not adjusted for clinical confounders such as age, injury level, or mechanical ventilation. Thus, the observed associations between autonomic function and outcome should be interpreted as exploratory. However, these regressions have been reported after adjustment for established outcome predictors as well, but these analyses run a risk of statistical overfitting. Fourth, neurological improvement at long-term follow-up was assessed retrospectively using all available medical records with varying follow-up time (8 [3–42] months). Nevertheless, outcome assessment followed a strict protocol to minimise variability in assessment. Lastly, only systemic physiological signals (HR and ABP) were analysed, while intraspinal pressure and spinal cord perfusion pressure, which may better capture intraspinal physiology and predict outcome after TSCI [5, 6, 29], were not available in this cohort.

## Conclusions

HRV and BRS appear altered after TSCI and may reflect sympathovagal imbalance with parasympathetic predominance rather than globally depressed autonomic function. Systemic autonomic indices seem to significantly vary depending on level and severity of injury and may capture distinct aspects of autonomic dysfunction beyond clinical grading, making these indices potential contributors to the bedside assessment. Increased knowledge about autonomic dysfunction in TSCI may enable more individualised haemodynamic treatment strategies. However, HRV, BRS, and SC demonstrated very limited predictive value for outcome following TSCI, suggesting that prognostic information may rather be captured by intraspinal indices. Prospective, multicentre studies with long-term outcome are warranted to validate these findings and to determine the predictive value of systemic and intraspinal SC to individualise NIC management and aid prognostication in TSCI.

## Supplementary information

Supplementary information

## Data Availability

Data are available upon reasonable request.

## Declarations

We confirm that the manuscript complies with all instructions to authors. We confirm that authorship requirements have been met and the final manuscript was approved by all authors. We confirm that this manuscript has not been published elsewhere and is not under consideration by another journal. We confirm using the reporting checklist STROBE.
