# Supplementary material for: Autonomic function and signal complexity reflect level and severity of traumatic spinal cord injury
Source: Spinal Cord. 2026 Oct 12;64(11):991–1001. doi: 10.1038/s41393-026-01285-z (PMC13731128; doi:10.1038/s41393-026-01285-z)
Supplement: Supplementary file 1 [file 41393_2026_1285_MOESM1_ESM.docx]

note

# Supplementary tables and figures

## Supplementary table 1. International Classification of Disease codes of considered patients

| **Code** | **Description** |
| --- | --- |
| **Cervical** | |
| S14.0 | Concussion and oedema of cervical spinal cord |
| S14.1 | Other and unspecified injuries of cervical spinal cord |
| S14.2 | Injury of nerve root of cervical spine |
| S19.7 | Multiple injuries of neck |
| S12.0 | Fracture of first cervical vertebra |
| S12.1 | Fracture of second cervical vertebra |
| S12.2 | Fracture of other specified cervical vertebra |
| S12.7 | Multiple fractures of cervical spine |
| **Thoracic** | |
| S24.0 | Concussion and oedema of thoracic spinal cord |
| S24.1 | Other and unspecified injuries of thoracic spinal cord |
| S24.2 | Injury of nerve root of thoracic spine |
| S22.0 | Fracture of thoracic vertebra |
| S22.1 | Multiple fractures of thoracic spine |
| **Lumbar** | |
| S34.0 | Concussion and oedema of lumbar spinal cord |
| S34.1 | Other injury of lumbar spinal cord |
| S34.2 | Injury of nerve root of lumbar and sacral spine |
| **Other** | |
| T02.0 | Fractures involving head with neck |
| T02.1 | Fractures involving thorax with lower back and pelvis |
| T06.0 | Injuries of brain and cranial nerves with injuries of nerves and spinal cord at neck level |
| T06.1 | Injuries of nerves and spinal cord involving other multiple body regions |
| G95.2 | Cord compression, unspecified |
| G82.0 | Flaccid paraplegia |
| G82.1 | Spastic paraplegia |
| G82.2 | Paraplegia, unspecified |
| G82.3 | Flaccid tetraplegia |
| G82.4 | Spastic tetraplegia |
| G82.5 | Tetraplegia, unspecified |

## Supplementary table 2. Exact p-values Spearman’s correlation: HRV, BRS, and SC vs. baseline characteristics and physiological variables

| Variable | SDNN | RMSSD | BRS | HR SC-index | MAP SC-index | SBP SC-index | DBP SC-index |
| --- | --- | --- | --- | --- | --- | --- | --- |
| Age (years) | 0.807 | ***0.000121*** | ***0.00617*** | 0.914 | 0.253 | 0.266 | 0.786 |
| Sex (female) | 0.439 | 0.522 | ***0.0322*** | 0.230 | 0.401 | 0.703 | 0.230 |
| CCI (scale) | 0.876 | ***5.196e-05*** | ***0.0246*** | 0.662 | 0.275 | 0.449 | 0.754 |
| Days in NIC (days) | ***0.0119*** | 0.630 | 0.937 | ***0.0402*** | 0.522 | 0.0945 | 0.898 |
| Mechanical ventilation during NIC (%) | 0.138 | 0.0621 | 0.126 | ***4.727e-11*** | ***0.000679*** | ***4.602e-06*** | ***0.0127*** |
| Vasopressor during NIC (yes) | ***0.00113*** | 0.892 | 0.558 | ***4.564e-06*** | 0.844 | 0.824 | 0.883 |
| Analgesics during NIC (yes) | 0.106 | 0.577 | 0.251 | 0.260 | 0.106 | 0.106 | 0.0932 |
| MAP (mmHg) | 0.954 | 0.543 | 0.133 | 0.846 | 0.127 | 0.112 | 0.207 |
| SBP (mmHg) | 0.180 | 0.196 | ***0.0388*** | 0.941 | 0.121 | ***0.0191*** | 0.276 |
| DBP (mmHg) | 0.699 | 0.284 | 0.108 | 0.389 | 0.0521 | 0.145 | 0.174 |
| HR (BPM) | ***1.179e-16*** | ***0.00157*** | ***3.077e-09*** | ***0.000951*** | 0.217 | 0.416 | 0.387 |

P-value below 0.05 was considered statistically significant. CCI = Charlson Comorbidity Index. NIC = neurointensive care. MAP = mean arterial pressure. SBP = systolic blood pressure. DBP = diastolic blood pressure. HR = heart rate. BPM = beats per minute. SDNN = standard deviation of all NN intervals. RMSSD = root mean square of adjacent NN interval differences. BRS = sequence baroreflex sensitivity. SC-index = signal complexity index. %VMT = percentage of valid monitoring time.

## Supplementary table 3. Univariable and multivariable binomial logistic regression: HRV, BRS, and SC vs. neurological improvement at discharge (yes/no) and mortality at 6 months (yes/no) of cervical injuries (n=138)

| Variable | Univariable | | | | Multivariable | | | |
| --- | --- | --- | --- | --- | --- | --- | --- | --- |
|  | Favourable outcome (improvement at discharge) | | Mortality (6 months) | | Favourable outcome (improvement at discharge) | | Mortality (6 months) | |
|  | OR (95% CI) | p-value | OR (95% CI) | p-value | OR (95% CI) | p-value | OR (95% CI) | p-value |
| SDNN (ms) | 0.99 (0.98–1.00) | 0.1480 | 1.00 (0.99–1.01) | 0.5840 | 0.99 (0.983–1.00) | 0.287 | 1.00 (0.99–1.01) | 0.584 |
| RMSSD (ms) | 0.99 (0.97–1.0) | 0.0647 | 1.01 (1.00–1.02) | ***0.0261*** | 0.99 (0.98–1.00) | 0.125 | 1.00 (0.99–1.01) | 0.953 |
| BRS (ms/mmHg) | 0.99 (0.88–1.09) | 0.801 | 1.04 (0.93–1.14) | 0.477 | 0.98 (0.86–1.09) | 0.687 | 0.98 (0.85–1.09) | 0.703 |
| HR SC-index | 1.09 (1.01–1.18) | ***0.0200*** | 0.97 (0.89–1.06) | 0.525 | 1.07 (0.99–1.17) | 0.104 | 0.98 (0.89–1.07) | 0.639 |
| MAP SC-index | 1.02 (0.92–1.13) | 0.766 | 1.05 (0.94–1.17) | 0.417 | 1.01 (0.90–1.13) | 0.908 | 1.06 (0.93–1.22) | 0.385 |
| SBP SC-index | 1.02 (0.93–1.12) | 0.659 | 0.97 (0.87–1.07) | 0.528 | 1.02 (0.92–1.14) | 0.701 | 0.95 (0.85–1.07) | 0.399 |
| DBP SC-index | 0.98 (0.88–1.09) | 0.713 | 1.03 (0.92–1.14) | 0.648 | 0.97 (0.87–1.08) | 0.574 | 1.04 (0.92–1.19) | 0.514 |

For the multivariable analyses, each row is a separate multivariable logistic regression for one predictor, adjusted for age (years), AIS at admission (grade), neurological level of injury (high cervical C1-4, low cervical C5-8), and percentage of neurointensive care time on mechanical ventilation. Odds ratios are presented per 1 unit. P-value below 0.05 was considered statistically significant. Missing: For improvement at discharge: 1. SDNN = standard deviation of all NN intervals. ms = milliseconds. RMSSD = root mean square of adjacent NN interval differences. BRS = sequence baroreflex sensitivity. HR = heart rate. SC-index = signal complexity index. MAP = mean arterial pressure. SBP = systolic blood pressure. DBP = diastolic blood pressure.

## Supplementary table 4. Multivariable binomial logistic regression: HRV, BRS, and SC vs. neurological improvement at discharge (yes/no) and mortality at 6 months (yes/no)

| Variable | Improvement at discharge (yes) | | Mortality at 6 months (yes) | |
| --- | --- | --- | --- | --- |
|  | OR (95% CI) | p-value | OR (95% CI) | p-value |
| SDNN (ms) | 1.00 (0.999–1.01) | 0.487 | 1.00 (0.99–1.01) | 0.425 |
| RMSSD (ms) | 0.99 (0.98–1.00) | 0.118 | 1.00 (0.99–1.01) | 0.935 |
| BRS (ms/mmHg) | 1.00 (0.90–1.10) | 0.943 | 0.97 (0.84–1.08) | 0.591 |
| HR SC-index | 1.06 (0.98–1.14) | 0.135 | 0.96 (0.87–1.04) | 0.314 |
| MAP SC-index | 1.03 (0.94–1.14) | 0.507 | 1.02 (0.90–1.16) | 0.774 |
| SBP SC-index | 1.05 (0.95–1.15) | 0.338 | 0.93 (0.83–1.04) | 0.201 |
| DBP SC-index | 1.01 (0.92–1.12) | 0.798 | 1.01 (0.89–1.13) | 0.927 |

Each row is a separate multivariable logistic regression for one predictor, adjusted for age (years), AIS at admission (grade), neurological level of injury (high cervical C1-4, low cervical C5-8, high thoracic Th1-6, low thoracic Th7-12), and percentage of neurointensive-care time on mechanical ventilation. Odds ratios are presented per 1 unit. P-value below 0.05 was considered statistically significant. Missing: For improvement at discharge: 1. SDNN = standard deviation of all NN intervals. ms = milliseconds. RMSSD = root mean square of adjacent NN interval differences. BRS = sequence baroreflex sensitivity. HR = heart rate. SC-index = signal complexity index. MAP = mean arterial pressure. SBP = systolic blood pressure. DBP = diastolic blood pressure.

## Supplementary figure 1. Selection of study population

All patients with ICD-codes corresponding to traumatic spinal cord injury, between 2008–2022 in the NIC unit at Uppsala University Hospital,

n=314

Patients with no actual spinal cord injury, n=112

Patients aged less than 18 years at injury time, n=10

Patients with invasive ABP monitoring less than 24h,

n=9

Patients with no invasive ABP monitoring, n=14

Patients with lumbar spinal cord injury, n=5

Included patients

n=164

Supplementary figure 1 presents the inclusion and exclusion process of the study population. A total of 314 patients treated in NIC at Uppsala University Hospital (2008–2022) with ICD-codes corresponding to spinal cord injury. Patients aged less than 18 years at injury time (n=10), patients with no actual traumatic spinal cord injury (n=112), patients with lumbar spinal cord injury (n=5), patients with no invasive ABP monitoring (n=14), and Patients with invasive ABP monitoring less than 24h (n=9) were excluded. Thus, the final cohort consisted of 164 patients.

## Supplementary figure 2. Density of heart rate variability, baroreflex sensitivity, and signal complexity

Density heatmaps show the normalised distribution of time spent within each variable range across patients. For each bin, %VMT was calculated per patient, across all patients, and normalised to a 0 (red) – 1 (blue) scale. SDNN = standard deviation of all NN intervals. RMSSD = root mean square of successive differences between adjacent NN intervals. BRS = baroreflex sensitivity. HR = heart rate. SC-index = signal complexity index. MAP = mean arterial pressure. SBP = systolic blood pressure. DBP = diastolic blood pressure. %VMT = percentage of valid monitoring time.

## Supplementary figure 3. GAM plots of raw haemodynamics vs. time stratified for cervical vs. thoracic surgeries

GAM plots for raw haemodynamic variables across the 7-day observation period. Blue represent patients with cervical injuries, and red patients with thoracic injuries. HR = heart rate. MAP = mean arterial pressure. SBP = systolic blood pressure. D
